# Protocol of a Multicenter Prospective Trial of Office-Based Carpal Tunnel Release With Ultrasound Guidance (ROBUST)

**DOI:** 10.7759/cureus.37479

**Published:** 2023-04-12

**Authors:** Ashley L Pistorio, Kevin C Chung, Larry E Miller, Julie E Adams, Warren C Hammert

**Affiliations:** 1 Department of Plastic Surgery, Kirk Kerkorian School of Medicine, University of Nevada, Las Vegas, USA; 2 Comprehensive Hand Center, Michigan Medicine, University of Michigan, Ann Arbor, USA; 3 Department of Biostatistics, Miller Scientific, Johnson City, USA; 4 Department of Orthopedic Surgery, University of Tennessee College of Medicine, Chattanooga, USA; 5 Department of Orthopedic Surgery, Division of Hand Surgery, Duke University Medical Center, Durham, USA

**Keywords:** ultrasound, ultraguide, robust, ctr-us, carpal tunnel syndrome, carpal tunnel release

## Abstract

Background

Carpal tunnel release (CTR) is a common surgical procedure for patients with severe or refractory carpal tunnel syndrome (CTS) symptoms. Historically, CTR procedures have been performed in a hospital or an ambulatory surgery center (ASC). However, due to advancements in techniques, greater patient demand, and concerns about growing healthcare costs, there is a distinct trend toward performing CTR procedures in an office-based setting. Several small studies with limited follow-up duration have demonstrated the feasibility of CTR with ultrasound guidance (CTR-US) when performed in an office-based setting. The objective of this study is to evaluate the safety and effectiveness of office-based CTR-US in a large cohort of patients (n=140) with symptomatic CTS followed for two years post-treatment.

Design and methods

ROBUST is a prospective multicenter observational study in which 140 subjects at up to 12 sites in the United States will be treated with CTR-US in an office-based setting. The primary endpoint of the study is the change in the Boston Carpal Tunnel Questionnaire Symptom Severity Scale score. Secondary endpoints include time to return to normal daily activities, time to return to work among employed subjects, change in the Boston Carpal Tunnel Questionnaire Functional Status Scale score, change in the Michigan Hand Questionnaire overall and domain scores, change in the Numeric Pain Scale score, change in the EuroQoL-5 Dimension 5-Level score, global satisfaction scores, and the incidence of device or procedure-related adverse events. The primary analysis of study endpoints will occur three months post-treatment. Patient follow-up in this study will continue for two years.

Conclusions

A central institutional review board approved the study protocol, and a data safety monitoring board will provide study oversight. The authors plan to report study results at medical conferences and in peer-reviewed medical journals. The outcomes of ROBUST will provide physicians, patients, and payors with important safety and effectiveness data regarding the clinical utility of CTR-US when performed in an office setting.

## Introduction

Carpal tunnel syndrome (CTS) is the most common peripheral compressive neuropathy and a leading cause of symptoms leading to presentation at a musculoskeletal or hand clinic [1]. Carpal tunnel release (CTR) is a reasonably safe and effective surgery that improves CTS symptoms by dividing the transverse carpal ligament (TCL) to relieve pressure on the median nerve [2]. While CTR procedures have been historically performed in a hospital-based operating room or ambulatory surgery center, advances in surgical techniques and anesthesia have allowed surgeons to perform the procedures in different operative settings. Because the operative setting is the primary cost driver of CTR [3], there has been recent interest in performing CTR in an office-based setting using local anesthesia, which may contribute to lower cost, faster patient recovery, greater patient satisfaction, and similar low complication rates [4-7].

Carpal tunnel release with ultrasound guidance (CTR-US) is well-suited as an office-based procedure because it typically uses a small (< 1 cm) incision to access and cut the TCL using continuous ultrasound guidance. Ultrasound helps the operator perform the procedure through a small incision while providing an expanded field of view, including multiplanar and dynamic views, compared to endoscopic and similar small incision techniques. Numerous studies have demonstrated the safety and effectiveness of CTR-US for the treatment of CTS [8-12], yet clinical outcomes with this procedure when performed in-office are not well characterized. A prospective single-center study demonstrated the feasibility of CTR-US when performed in an office-based setting [8]. The improvement in CTS symptoms following office-based CTR-US was statistically significant and clinically important over one year of follow-up, and no intraoperative complications or reoperations were reported. However, it remains unclear whether these results are reproducible when treating patients with office-based CTR-US across multiple clinical practices. Thus, the objective of this prospective multicenter, observational study is to evaluate the safety and effectiveness of office-based CTR-US in a large cohort of patients with symptomatic CTS followed for two years post-treatment.

## Materials and methods

This paper describes the rationale and design of ROBUST (a multicenter prospective trial of office-based carpal tunnel release with ultrasound guidance). The study protocol follows the SPIRIT 2013 (Standard Protocol Items: Recommendations for Interventional Trials) guidance for protocols of clinical trials [13].

Study design

This is a prospective, multicenter, observational study performed at up to 12 sites in the United States, with subject recruitment starting in Q1 2023. A total of 140 subjects will be enrolled and treated with unilateral or bilateral simultaneous CTR-US. The total study duration is expected to be approximately 2.5 years, with six months of anticipated subject recruitment and two years of follow-up. We prospectively registered the trial at ClinicalTrials.gov (NCT05675046) before the first subject was enrolled. Sonex Health, Inc. (Eagan, MN) provided funding for the trial. The study sponsor participated in the design of the trial but will not be involved in data analysis or the decision to submit trial results for publication. A data safety monitoring board (DSMB) will provide study oversight.

Participants and eligibility criteria

All patients presenting with refractory, idiopathic CTS who are determined to be candidates for CTR will be considered for enrollment. Patients and treating surgeons will discuss surgical options for CTR, including CTR-US, through a shared decision-making process. Patients choosing CTR-US will undergo a preoperative clinical examination with diagnostic ultrasound of the median nerve. Key study eligibility criteria are a clinical diagnosis of unilateral or bilateral idiopathic CTS, CTS-6 score ≥ 12 in the target hand(s) [14], median nerve cross-sectional area ≥10 mm2 at the carpal tunnel inlet region of the target hand(s) [14,15], and persistent symptoms following nonsurgical CTS treatment. Key exclusion criteria are previous CTR or infection in the target hand, recent (<6 weeks) corticosteroid injection on the target wrist or hand, or planned surgical or interventional procedure on the contralateral wrist or hand within three months. Subjects who meet all preoperative eligibility criteria will be scheduled to receive CTR-US. A complete list of study eligibility criteria is provided in Table 1.

**Table 1:** Subject eligibility criteria *Criterion must be applied to the target hand for unilateral procedures or to both hands for simultaneous bilateral procedures. **Clinically significant is defined as likely to interfere with the performance of the procedure in a safe and/or effective manner. CTR, carpal tunnel release; CTS, carpal tunnel syndrome

Inclusion Criteria
≥18 years of age
Clinical diagnosis of unilateral or bilateral idiopathic CTS
CTS-6 score >12 in target hand*
Median nerve cross-sectional area ≥10 mm2 in the proximal carpal tunnel region of the target hand measured by diagnostic ultrasound*
Prior failure of one or more nonsurgical treatment options (e.g., physical activity modification, bracing, splinting, corticosteroid injection)*
Subject agrees to complete follow-up questionnaires over a 24-month period
Subject has a valid smartphone number and/or email address to receive and answer follow-up questionnaires
Exclusion Criteria
Prior surgery on the target wrist or hand with the exception of (a) trigger finger release or similar minor finger procedure (e.g., digital ganglion cyst removal, foreign body removal) that has clinically recovered, or release for DeQuervain’s syndrome (1^st^ dorsal compartment) that has clinically recovered*
History of prior surgical CTR in the target hand*
History of infection in the target hand*
History of prior surgery in the non-target hand, including CTR, within 3 months of enrollment or with persistent symptoms that interfere with normal daily activities or work at the time of consent
Planned surgical or interventional procedure on the contralateral hand within 3 months of the target hand procedure date*
Corticosteroid injection in the target hand within 6 weeks of the study procedure date*
Presence of additional process in the target hand requiring additional intervention beyond carpal tunnel release (e.g. neurolysis, mass removal, tenosynovectomy)*
Clinically significant** degenerative arthritis of the upper limb (shoulder to hand) on the target side*
Clinically significant** inflammatory disease (including tenosynovitis) of the upper limb (shoulder to hand) on the target side
Clinically significant** trauma or deformity of the upper limb (shoulder to hand) on the target side*
Clinically significant** vascular disease (including Raynaud’s phenomenon) of the upper limb (shoulder to hand) on the target side*
Clinically significant** neurological disorder (including complex regional pain syndrome) of the upper limb (shoulder to hand) on the target side*
Systemic inflammatory disease (e.g., rheumatoid arthritis, lupus)
Amyloidosis
Chronic renal insufficiency requiring dialysis
Diabetes not controlled by a stable dose of medication
Uncontrolled thyroid disease
Pregnant or planning pregnancy in the next 24 months
Workers’ compensation subjects
Inability to provide a legally acceptable Informed Consent Form and/or comply with all follow-up requirements
Subject has other medical, social, or psychological conditions that, in the opinion of the investigator, preclude them from receiving the pre-treatment, required treatment, and post-treatment procedures and evaluations

Surgical procedure

All CTR-US procedures will be performed in-office. Per the Place of Service Codes set forth by the Centers for Medicare & Medicaid Services (CMS), in-office is defined as a location, other than a hospital, skilled nursing facility, military treatment facility, community health center, state or local public health clinic, or intermediate care facility, where the health professional routinely provides health examinations, diagnosis, and treatment of illness or injury on an ambulatory basis [16].

Subjects will be treated with CTR-US using the commercially available UltraGuideCTR® (Sonex Health, Inc., Eagan, MN). The device is a single-use, hand-held device that is inserted into the carpal tunnel through a small (<1 cm) incision at the proximal wrist using real-time ultrasound guidance. The working tip consists of a blunt dissecting uniquely echogenic tip with a working shaft that houses two inflatable balloons that border a centrally located, retractable retrograde cutting blade. Ultrasound is used to aid in positioning the tip deep and distal to the TCL and the balloons are inflated with sterile saline to create and maintain space within the carpal tunnel. Ultrasound is then used to confirm the safe positioning of the working tip relative to the surrounding anatomy. The blade is then activated, and the TCL is transected in a retrograde manner under continuous ultrasound guidance. Following TCL transection, the blade is recessed, the balloons are deflated, locking the blade in position, and the device is removed. The TCL is then probed under ultrasound guidance to ensure a complete release has been performed.

Postoperative patient care instructions will be standardized among all participating sites in order to minimize bias owing to the known association of physician recommendations with return to work after CTR [17]. Investigators will instruct subjects to participate in activities and return to work, as tolerated, based on pain, function, and wound healing status.

Outcomes

Postoperative subject data will be obtained remotely using an electronic data capture system that will be monitored for completeness and accuracy throughout the study. Subject follow-up assessments will occur daily for the first 14 post-procedure days, and at one, three, six, 12, and 24 months thereafter. Time to return to normal activities and return to work will be assessed daily for the first 14 days, and at routine follow-up intervals. The Boston Carpal Tunnel Questionnaire Symptom Severity Scale (BCTQ-SSS) and Functional Status Scale (BCTQ-FSS) scores, Michigan Hand Questionnaire (MHQ) scores, Numeric Pain Scale scores, EuroQoL-5 Dimension 5-Level (EQ-5D-5L) scores, global satisfaction responses, and adverse events (AEs) will be assessed at each follow-up interval. A schedule of subject assessments during the study is provided in Table 2.

**Table 2:** Study assessments at each follow-up interval *Performed on all treated hands. **Measured on both hands. ***Collected at 14-day evaluation only. †Collected on employed subjects only. BCTQ-FSS, Boston Carpal Tunnel Questionnaire Functional Status Scale; BCTQ-SSS, Boston Carpal Tunnel Questionnaire Symptom Severity Scale; EQ-5D-5L, EuroQoL-5 Dimension 5-Level; MHQ, Michigan Hand Questionnaire.

Assessment	Baseline	Procedure	Post-Op	Daily 1-14	Months				
					1	3	6	12	24
Site Assessments									
Demographics	■								
Ultrasound median nerve cross-sectional measurement	■ *								
CTS-6	■ **								
Procedure		■							
Adverse events		■	■	■	■	■	■	■	■
Subject-reported outcomes									
Demographics	■								
Medical history	■								
BCTQ-SSS	■			■ ***	■	■	■	■	■
BCTQ-FSS	■			■ ***	■	■	■	■	■
MHQ	■			■ ***	■	■	■	■	■
Numeric Pain Scale	■	■	■	■	■	■	■	■	■
EQ-5D-5L	■			■ ***	■	■	■	■	■
Global satisfaction				■ ***	■	■	■	■	■
Procedure		■							
Return to activities				■	■	■	■	■	■
Return to work †				■	■	■	■	■	■
Pain medication	■	■	■	■	■	■	■	■	■

The primary endpoint of the study is the change in BCTQ-SSS score at three months compared to baseline. The BCTQ is a CTS-specific questionnaire that is highly reproducible, internally consistent, valid, and responsive to clinical change in CTS and subject status post-CTR [18]. The BCTQ consists of 11 symptom severity questions (BCTQ-SSS) that are scored from 1 to 5, with higher scores indicating more severe symptoms, and is calculated as the mean of each response.

Secondary endpoints of the study are the time to return to normal daily activities, time to return to work among employed subjects, change in BCTQ-FSS score at three months compared to baseline, change in MHQ overall and domain scores at three months compared to baseline, change in Numeric Pain Scale score at three months compared to baseline, change in EQ-5D-5L score at three months compared to baseline, global satisfaction score at three months, and the incidence of device- or procedure-related AEs at three months. The three-month follow-up interval was selected for the initial analysis of endpoints because the majority of clinical improvement after CTR occurs in the first three postoperative months [19,20]. Patient follow-up data will be collected for 24 months post-treatment to determine procedure durability and long-term safety results.

Time to return to normal daily activities is defined as the number of days between CTR-US and when the subject reports returning to normal daily activities outside of work. Among employed subjects, time to return to work is defined as the number of days between CTR-US and when the subject reports returning to work in any capacity. The BCTQ-FSS consists of eight functional status questions that are scored from 1 to 5, with higher scores indicating more functional limitation, and is calculated as the mean of each response. The MHQ is a validated, hand-specific questionnaire consisting of 37 questions across six domains including overall hand function, activities of daily living, work performance, pain, aesthetics, and satisfaction [21]. The MHQ provides an overall score as well as scores for each domain ranging from 0 to 100 where higher values represent better outcomes, with the exception of the pain domain where lower values represent less pain. Subjects will rate wrist pain severity on a Numeric Pain Scale ranging from 0 to 10, where 0 represents “no pain” and 10 represents “the worst pain imaginable.” The EQ-5D-5L is a generic preference-based questionnaire that measures the quality of life across five domains: mobility, self-care, usual activities, pain/discomfort, and anxiety/depression. Each dimension is scored on a five-level severity ranking consisting of no problems, slight problems, moderate problems, severe problems, and unable to/extreme problems [22]. Global satisfaction will be determined by asking subjects to rate their satisfaction with the procedure using a five-point Likert scale ranging from very dissatisfied to very satisfied, as well as asking how likely they are to recommend the procedure to a friend or colleague using a five-point Likert scale ranging from very unlikely to very likely.

Subject safety will be assessed by recording AEs occurring after the initial procedural incision has been initiated. Determination of whether a subject experienced an AE may be made in two different ways. First, an AE can be documented by the site during the study procedure. Second, a subject may report a postoperative AE directly via phone call to the investigative site.

Adverse events will be classified by seriousness and relationship to the device or procedure. A serious adverse event (SAE) is defined as one that suggests a significant hazard or side effect, regardless of the relationship to the device or procedure. A device-related AE is directly attributable to the device or to improper use of the device. A procedure-related AE is directly attributable to the procedure, irrespective of the device, including complications from anesthesia or other procedures incidental to CTR-US. The relationship of the AE to the device or procedure will be determined by the site investigator and classified as “definite”, “probable”, “possible”, “unlikely”, or “not related.”

Evaluation and adjudication of all AEs will be performed by an independent medical reviewer. The independent medical reviewer will review AEs for AE classification, seriousness, and relationship with the device or procedure. Discrepancies between the investigational site and the independent medical reviewer will be handled by discussion, with the determination of the independent medical reviewer serving as the final classification.

A DSMB will oversee the enrollment and safety of the study subjects and will advise the study sponsor to continue the study with no modification or to modify the study as appropriate if enrollment or safety concerns are identified. Members of the DSMB will be independent of the sponsor and investigational sites.

Statistical analysis

A power analysis determined that 100 subjects provided 90% statistical power to detect a change of at least 0.2 points on the BCTQ-SSS at three months assuming a standard deviation of 0.75 points and a two-tailed alpha level of 0.05. To account for reasonable subject attrition, a minimum of 140 subjects will be enrolled in the study.

The analysis population will consist of all enrolled subjects who receive their assigned treatment. Baseline data will be analyzed using mean and standard deviation for normally distributed data, the median and interquartile range for non-normally distributed data, and counts and percentages for categorical data. The primary endpoint of BCTQ-SSS change at three months will be analyzed using a mixed model analysis. Time to return to normal daily activities and time to return to work among employed individuals will be reported as the median and interquartile range in each treatment group. Longitudinally measured continuous outcomes will be analyzed using mixed model analysis. Adverse events will be reported using counts, percentages, and exact 95% confidence intervals. Statistical analyses will be performed using a two-sided hypothesis test at a 5% level of significance.

Ethics and dissemination

A central institutional review board (WCG IRB, Puyallup, WA) approved the study protocol and subjects will provide written informed consent before study participation. The authors plan to disseminate the short-, mid-, and long-term results of this study at major medical conferences and submit them for publication to peer-reviewed journals.

## Results

The results of the ROBUST study are expected in Q2 2024 and will fill an important research gap in the existing CTR literature.

## Discussion

Carpal tunnel release is a common surgery with approximately 600,000 procedures performed each year in the United States alone [1,23]. However, only 2% of these procedures are currently performed in an office setting [7]. Potential concerns regarding transferring procedures from the operating room to the office include the risk of higher complication rates and poorer outcomes. However, current evidence suggests that these concerns may be unfounded [4-7]. Given the efforts to improve value in health care, further investigation is warranted to understand the association between operative settings and patient outcomes with CTR.

The ROBUST study is novel because all patients will be treated with CTR-US in an office setting. The trial design is rigorous with a large cohort of patients recruited from multiple centers across the United States who will be followed for two years after surgery. The main limitation of the study is that it is observational in nature with no control group. We plan to report the three-month results of the study in Q2 2024, which will include important metrics such as return to normal activity, return to work, patient-reported clinical outcomes, and complication rates.

## Conclusions

This paper describes the protocol for the ROBUST study. The results of the ROBUST study will fill an important research gap in the existing CTR literature by providing physicians, patients, and payors with the long-term results of CTR-US when performed in an office setting.
